# Genome Sequence of *Penicillium solitum* RS1, Which Causes Postharvest Apple Decay

**DOI:** 10.1128/genomeA.00363-16

**Published:** 2016-05-12

**Authors:** Jiujiang Yu, Guangxi Wu, Wayne M. Jurick, Verneta L. Gaskins, Yanbin Yin, Guohua Yin, Joan W. Bennett, Daniel R. Shelton

**Affiliations:** a USDA-ARS, Beltsville Agricultural Research Center, Beltsville, Maryland, USA; b Oak Ridge Institute for Science and Education, Oak Ridge, Tennessee, USA; c Department of Biological Sciences, Northern Illinois University, DeKalb, Illinois, USA; d Department of Plant Biology and Pathology, Rutgers University, New Brunswick, New Jersey, USA

## Abstract

*Penicillium* species cause postharvest decay, commonly known as blue mold, in pome fruits, such as apples and pears. To devise novel strategies to prevent and reduce economic losses during storage, the genome sequence of *Penicillium solitum* RS1 is reported here for the first time.

## GENOME ANNOUNCEMENT

*Penicillium* is a fungal genus composed of saprophytes and human pathogens and includes several plant-pathogenic species that infect pome fruits (1). *Penicillium solitum* RS1, isolated from decayed apples during storage in Oregon, is a less virulent species than *Penicillium expansum* (2). To deepen our understanding of the genetic differences likely contributing to their virulence, spore germination, and mycotoxin production, the genome of the wild-type strain of *P. solitum* (RS1) was sequenced, assembled, and annotated for comparative genomics study with the more virulent species *P. expansum* R19 strain (2).

Spores of *P. solitum* RS1 were inoculated in potato dextrose broth (PDB) medium at a final concentration of 10^6^/ml and incubated at 25°C for 7 days under constant shaking at 150 rpm in a temperature-controlled incubator. Genomic DNA was prepared with the DNeasy plant maxi kit (Qiagen), according to the manufacturer’s instructions. Single-molecule real-time (SMRT) sequencing reads were generated using the PacBio RSII sequencer. The sequence depth reached 60.54×. All reads were used to generate assemblies with HGAP3 under default conditions. The final assembly contains 35.8 Mbp in seven scaffolds, with an *N*_50_ value of 7.7 Mbp. This is comparable with the genome sizes reported previously for *P. expansum* R19 (2) and *Penicillium chrysogenum* (3).

The genome sequence of *P. solitum* (RS1) was annotated using the MAKER program (4) in an iterative fashion, for a total of four runs. Core eukaryotic genes were first predicted using CEGMA (5) and were used to train the *ab initio* predictors AUGUSTUS (6) and SNAP (7). Protein evidence from several related species, including *Aspergillus fumigatus* (8), *Aspergillus oryzae* (9), *P. expansum*, and *P. chrysogenum* (3), was also used. The results of the previous annotation were used to retrain the two *ab initio* predictors for the next annotation analysis. A total of 10,672 coding genes were annotated in the *P. solitum* (RS1) genome.

The predicted genes were then annotated with known protein domains using Pfam. A total of 8,075 proteins were annotated via 3,899 Pfam domains. The antiSMASH program (10) predicted 66 secondary metabolite biosynthetic gene clusters. Additionally, using MCL (11) clustering with default settings, unique gene sets in *P. solitum* and *P. expansum* were identified. There are about 285 unique genes in *P. solitum* RS1 that do not exist in *P. expansum* R19, while there are 224 genes that are exclusive to *P. expansum* (R19). Further investigation is needed to pinpoint genes that are putatively involved in various aspects of virulence, thus providing valuable information for devising novel approaches to combat fungal infection in pome fruits.

### Nucleotide sequence accession numbers.

This whole-genome shotgun project of the *P. solitum* RS1 has been deposited at DDBJ/EMBL/GenBank under the accession no. JYNM00000000. The version described in this paper is version JYNM02000000.
